# LITAF suppresses breast cancer and paclitaxel resistance by ubiquitinating and degrading PCMT1 to inhibit COX-2-dependent arachidonic acid metabolism

**DOI:** 10.3389/fphar.2026.1706420

**Published:** 2026-04-13

**Authors:** Na Yang, Jingying Cao, Feng Jiang, Renxian Cao, Yiqi Liu

**Affiliations:** 1 The First Affiliated Hospital, Gynecology & Obstetrics and Reproductive Medical Center, Hengyang Medical School, University of South China, Hengyang, China; 2 Department of Medicine Clinical Laboratory, The Third Xiangya Hospital of Central South University, Changsha, China; 3 Institute of Clinical Medicine, The First Affiliated Hospital, Hengyang Medical School, University of South China, Hengyang, China

**Keywords:** AA metabolism, breast cancer, LITAF, PCMT1, PTX-resistance

## Abstract

**Background:**

Paclitaxel (PTX) is a first-line chemotherapeutic agent extensively employed in the management of breast cancer (BC); however, the emergence of drug resistance frequently results in unsatisfactory clinical outcomes and poor prognosis. This study aimed to investigate the pathogenic mechanisms that drive PTX resistance in BC.

**Methods:**

Tumor and matched adjacent normal tissues were collected from 30 BC patients treated with PTX. Untargeted metabolomics was performed to analyze the metabolic differences. The expression of lipopolysaccharide-induced tumor necrosis factor-alpha factor (LITAF), protein L-isoaspartyl (D-aspartyl) methyltransferase (PCMT1), and cyclooxygenase-2 (COX-2) was assessed using RT-qPCR, immunoblotting, and immunohistochemistry (IHC). Cell proliferation was determined via CCK-8 and colony formation assays, cell apoptosis was analyzed by flow cytometry, and enzyme-linked immunosorbent assay (ELISA) was used to measure arachidonic acid (AA) and prostaglandin E2 levels. The interaction between LITAF and PCMT1, as well as the ubiquitination level of PCMT1, was investigated using co-immunoprecipitation (Co-IP). *In vivo*, nude mice were used to explore the effect of LITAF on tumor response to PTX treatment.

**Results:**

PCMT1 and COX-2 were upregulated in BC tissues, particularly in PTX-resistant patients, whereas LITAF expression was downregulated. In BC tissues, LITAF expression was negatively correlated with PCMT1 levels, while PCMT1 expression showed a positive correlation with COX-2 levels. PCMT1 knockdown attenuated COX-2-mediated AA metabolism, suppressed BC cell proliferation, and increased the sensitivity of BC cells to PTX. LITAF interacted with PCMT1 and promoted ubiquitination-mediated degradation of PCMT1, thereby inhibiting COX-2-mediated AA metabolism, reducing the proliferation of PTX-resistant BC cells, and enhancing the sensitivity of BC cells to PTX *in vivo*.

**Conclusion:**

LITAF regulates the ubiquitination-mediated degradation of PCMT1 to inhibit COX-2-dependent AA metabolism, thereby enhancing the sensitivity of BC cells to PTX and providing a potential therapeutic strategy to overcome PTX resistance in BC.

## Highlight

- Inhibition of PCMT1 weakened COX-2-mediated AA metabolism in BC cells.
- PCMT1 knockdown restrained BC proliferation and enhanced the sensitivity of BC cells to PTX by modulating the AA pathway.
- LITAF interacted with PCMT1 and regulated the ubiquitination-dependent degradation of PCMT1.
- LITAF modulated PCMT1-mediated AA metabolism to suppress the proliferation of PTX-resistant BC cells and enhanced BC cells' sensitivity to PTX *in vivo.*

## Introduction

The 2022 global cancer statistics showed that breast cancer (BC) accounts for 11.6% of all cancers worldwide, making it the most common malignancy in women and the main contributor to female cancer-related mortality (Bray et al., 2024). Its incidence has been increasing annually, making it a serious and continuously escalating threat to patients’ health (Yuan et al., 2024). Current treatment options for BC encompass surgical intervention, radiotherapy, cytotoxic chemotherapy, hormone therapy, molecularly targeted agents, and immunotherapy, and together these modalities have significantly advanced BC treatment by providing patients with more personalized and, in many cases, less invasive therapeutic strategies (Gu et al., 2024; Loibl et al., 2021). Despite these advances, treatment outcomes still vary considerably among individuals, reflecting marked heterogeneity in tumor biology and treatment response (Song et al., 2024). Paclitaxel (PTX) is an important chemotherapeutic agent in BC management and is widely used in clinical practice (Skubnik et al., 2023; Zhou et al., 2023). It is also one of the primary systemic therapies for triple-negative breast cancer (Wang et al., 2025) and constitutes an important component of adjuvant regimens for high-risk estrogen receptor (ER)-positive disease, particularly in patients with advanced ER-positive BC (Saji et al., 2022; Turner et al., 2019; Zhu et al., 2021; Masuda et al., 2018; Jiang et al., 2024). Although chemotherapy can improve prognosis and overall survival, intrinsic or acquired resistance to PTX remains a major challenge in BC treatment (Zhu et al., 2023; Liu et al., 2023). Therefore, identifying novel therapeutic targets that drive BC progression and PTX chemoresistance remains an urgent priority.

Protein L-isoaspartyl (D-aspartyl) methyltransferase (PCMT1) is an enzyme that repairs proteins containing altered aspartate residues through methylation, thereby helping to restore their native conformation and function (Liu et al., 2024). Our preliminary studies revealed that PCMT1 is overexpressed in BC tissues and correlates with overall survival, and that PCMT1 knockdown markedly reduced the metastatic potential of BC cells and significantly suppressed tumor growth (Liu et al., 2024). In line with these observations, previous reports have shown that inhibition of PCMT1 not only decreases the proliferation of BC cells (Saiding et al., 2023), but also induces apoptosis (Zhang et al., 2023). Recent studies further suggest that PCMT1 may be involved in PTX resistance in BC (Zhang et al., 2024), although the underlying molecular mechanisms remain to be fully elucidated.

Ubiquitination is a post-translational modification in which ubiquitin molecules are covalently attached to target proteins to direct their proteasomal degradation, a regulatory process that is critical in diverse physiological and pathological events in BC (Han et al., 2023). E3 ubiquitin ligases are key determinants of substrate specificity within the ubiquitin-proteasome degradation pathway. For instance, the E3 ubiquitin ligase FBXW2 suppresses BC progression and regulates PTX resistance by mediating the ubiquitination and degradation of p65 (Ren et al., 2022), while TRIM22 exerts a tumor-suppressive role in BC by targeting CCS for ubiquitin-dependent degradation (Yang et al., 2024). Lipopolysaccharide‐induced tumor necrosis factor‐alpha factor (LITAF) is an E3 ubiquitin ligase that regulates downstream biological functions by mediating the ubiquitination and degradation of its substrates (Min et al., 2025). It has been reported that LITAF exhibits anti-tumor effects in colorectal and prostate cancers (Guan et al., 2023; Zhou et al., 2011). In BC, LITAF activation has been suggested to contribute to LINC00173 silencing and to estrogen-mediated suppression of tumorigenesis (Xie et al., 2024). Notably, the Ubibrowser 2.0 database predicts LITAF as a potential E3 ubiquitin ligase for PCMT1; however, whether LITAF regulates BC progression and PTX chemoresistance through PCMT1 remains unknown.

Cyclooxygenase‐2 (COX-2) is a key enzyme in arachidonic acid (AA) metabolism, and COX-2-driven metabolic pathways contribute to the progression of multiple cancers, including colon and head and neck cancers (Zhang et al., 2025; Koontongkaew et al., 2010). COX-2 also plays an important role in BC development; for example, the COX-2 inhibitor celecoxib has been reported to enhance BC cell death by downregulating the E-cadherin complex (Balamurugan et al., 2023), while COX-2 overexpression attenuates the inhibitory effect of tamoxifen on BC cells (Tari et al., 2005). In ovarian cancer, the proportion of COX-2-positive cases is significantly higher in PTX non-responders than in PTX-sensitive patients (Ferrandina et al., 2006), suggesting that COX-2-mediated AA metabolism may also contribute to BC progression and PTX resistance.

This study aims to determine whether LITAF can weaken COX-2-mediated AA metabolism by regulating the ubiquitination and degradation of PCMT1 to limit BC progression and enhance sensitivity to PTX. Using clinical specimens, *in vitro* cellular models and *in vivo* animal experiments, we investigate the role of LITAF in BC tumor growth and PTX to identify potential diagnostic and therapeutic targets for BC patients, particularly those with PTX-resistant disease.

## Methods

### Clinical sample

This study included 30 patients diagnosed with BC who underwent neoadjuvant chemotherapy with PTX. Based on their response to PTX treatment, the patients were allocated into two groups: PTX-sensitive and PTX-resistant. Treatment response was evaluated using a combination of radiographic and pathological criteria. For radiographic assessment, tumor shrinkage was evaluated by magnetic resonance imaging (MRI), and tumor diameters were measured according to the RECIST version 1.1 guidelines (Buisseret et al., 2023). Pathological assessment was performed by experienced pathologists who examined post-treatment lesion tissues. PTX-sensitive disease was defined as the achievement of either a radiographic complete response (CR; disappearance of all target lesions) or partial response (PR; ≥30% reduction of target lesion diameters) (Zhang et al., 2020), or a major pathological response (MPR; ≤10% residual viable tumor cells). The remaining patients were classified as PTX-resistant. Among the 30 patients, 17 were PTX-resistant and 13 were PTX-sensitive. The collected tissue specimens were either cryopreserved at −80 °C or paraffin-embedded. Written informed consent was obtained from all participants, and the study protocol was approved by the Ethics Committee of The First Affiliated Hospital of University of South China.

### Cell culture, drug-resistant strain construction, and cell transfection

Human normal mammary epithelial cells MCF10A (ATCC, USA) were cultured in DMEM/F12 (Procell) supplemented with HS (5%), EGF (20 ng/mL), hydrocortisone (0.5 μg/mL), insulin (0.5 μg/mL), NEAA (1%), and P/S (1%). MDA-MB-231 (ATCC) was maintained in Leibovitz’s L-15 (Procell) containing FBS (10%) and P/S (1%). MCF-7 (ATCC) was maintained in MEM (Procell) supplemented with NEAA (1%), insulin (10 μg/mL), FBS (10%), and P/S (1%).

To generate PTX-resistant subpopulations, BC cells were continuously exposed to increasing concentrations of PTX for over 6 months (Min et al., 2022). To assess the responsiveness of cells to PTX, MDA-MB-231 was treated with 0, 0.01, 0.05, 0.1, 0.5, and 1 μM PTX for 48 h, while MCF-7 was treated with 0, 0.5, 1, 2, 5, and 10 μM PTX for 48 h, with DMSO serving as the control.

For the knockdown and overexpression of PCMT1, as well as the overexpression of LITAF, BC cells were transfected with sh-PCMT1, LITAF, or PCMT1 vectors (Genepharma, China), or their corresponding control vectors (sh-NC, vector) using Lipofectamine 3000 (ThermoFisher, USA) for 48 h. For treatment with MG132, MDA-MB-231 and MCF-7 in the various intervention groups were exposed to MG132 (10 μM, Sigma-Aldrich, Germany) for 4 h.

### Xenograft tumor model

First, MDA-MB-231/PTX was transduced with LITAF-overexpressing lentivirus (LITAF, GenePharma) or the corresponding control lentivirus (Vector) for 48 h, followed by selection with puromycin for 2 weeks to establish a stable LITAF-overexpressing MDA-MB-231/PTX cell line. BALB/c nude mice (female, 1–2 months, Slac Jingda, China) were stochastically allocated into four cohorts: Vector, LITAF, Vector + PTX, LITAF + PTX. In the Vector group, mice were subcutaneously inoculated with MDA-MB-231/PTX cells that had been stably transduced with Vector; in the LITAF group, mice received subcutaneous inoculations of MDA-MB-231/PTX that stably overexpressed LITAF; in the PTX-treated groups (Vector + PTX and LITAF + PTX), PTX (10 μg/g, Selleck, three times per week) was injected starting 1 week after cell inoculation (Ren et al., 2022). Tumor volume was assessed at weekly intervals. Following a 4-week experimental period, mice were humanely sacrificed, and tumor tissues were collected. All procedures involving animal subjects were approved by the Animal Ethics Committee.

### Immunohistochemistry (IHC)

Tumor specimens were fixed in 4% formalin for 24 h, embedded in paraffin, and sectioned into 5 μm-thick slices using a microtome. The sections were then subjected to deparaffinization in xylene, rehydration through a graded ethanol series, heat-induced epitope retrieval (HIER) using citrate buffer, and quenching of endogenous peroxidase activity with 3% H2O2. After washing, the sections were incubated with BSA (5%, Solarbio, China) for 30 min, followed by sequential incubation with primary antibodies anti-Ki67 (1:1000, ab15580, Abcam), overnight at 4 °C, and then with secondary antibody (1:1000, ab6721, Abcam) for 30 min. Lastly, staining was developed with 3,3′-diaminobenzidine (DAB) and counterstained with hematoxylin, and images were captured under a microscope (Olympus, Japan). Quantitative analysis was performed using ImageJ software.

### Untargeted metabolomics

After transfection with sh-NC or sh-PCMT1, MDA-MB-231 cells were collected, and ice-chilled 80% methanol was added to facilitate metabolite extraction, followed by agitation. The resulting lysate was maintained at −20 °C for 30 min to promote protein precipitation and subsequently centrifuged (20,000 × g, 4 °C) for 10 min. The supernatant was transferred to a new microcentrifuge tube and dried completely under vacuum. The dried metabolite extract was reconstituted in ice-cold 80% methanol and then transferred into an LC-MS vial equipped with a 200 μL insert. Chromatographic separation was performed on a Vanquish Flex UHPLC system (ThermoFisher) using an ACQUITY UPLC T3 column (100 mm × 2.1 mm, 1.7 µm) for reversed-phase separation. Metabolites eluting from the column were analyzed on a Q-Exactive mass spectrometer (ThermoFisher). The acquired data were processed using MSConvert and metaX for subsequent statistical analysis.

### Real-time quantitative polymerase chain reaction (RT-qPCR)

Total RNA was isolated and subsequently reverse-transcribed into cDNA for RT-qPCR using a TaqMan One Step RT-qPCR Kit (Solarbio) on a LightCycler® 96 real-time PCR system (Roche Diagnostics GmbH, Germany) to determine PCMT1 and LITAF expression levels. The 2^−ΔΔCT^ method was applied for gene expression quantification, with GAPDH used for normalization. The primer sequences employed were (5′-3′): PCMT1 forward (ATG​ATG​CCA​TTC​ATG​TGG​GAG) and PCMT1 reverse (GGA​CCA​ACA​GGC​AAT​ATC​AAT​CT), LITAF forward (ATG​TCG​GTT​CCA​GGA​CCT​TAC) and LITAF reverse (TAC​GAA​GGA​GGA​TTC​ATG​CCC), GAPDH forward (ACA​ACT​TTG​GTA​TCG​TGG​AAG​G) and GAPDH reverse (GCC​ATC​ACG​CCA​CAG​TTT​C).

### Immunoblotting

Total protein was extracted from cells/tissues using RIPA lysis buffer (Solarbio) and quantified using a BCA Protein Assay Kit (Solarbio). Protein lysates from different groups were separated by SDS-PAGE and electrophoretically transferred to PVDF membranes, which were blocked with 5% skim milk for 1 h, followed by 12 h incubation with specific primary antibodies at 4 °C and 1 h incubation with secondary antibody (1:3000, ab6721, Abcam). Protein bands were visualized using a Bio-Rad imaging system (USA) with an ECL Kit (Solarbio) and analyzed using ImageJ software. The primary antibodies, anti-PCMT1 (1:3000, ab97446), anti-COX-2 (1:5000, ab179800), anti-LITAF (1:2000, ab187533), anti-Ubiquitin (1:5000, ab134953), and anti-GAPDH (1:2000, ab9485) were obtained from Abcam.

### Enzyme-linked immunosorbent assay (ELISA)

Prostaglandin E2 (PGE2) and AA levels were measured using the PGE2 ELISA Kit (ab316263) and AA ELISA Kit (ab287798) from Abcam, respectively, following the manufacturer’s instructions.

### Cell viability and colony formation assays

For cell viability, the culture supernatant was removed from each well, followed by the addition of Cell Counting Kit-8 (CCK-8, 10 μL, Solarbio) and fresh medium (90 μL) per well. After 60 min of incubation, the optical density at 450 nm (OD450) was measured using a microplate reader (Molecular Devices, USA).

For colony formation, cells from each group were plated in 6-well plates and cultured in complete growth medium for 14 days. The resultant colonies were fixed with 4% paraformaldehyde, gently washed, stained with crystal violet (0.5%; Solarbio, China), and then washed again to remove excess dye. The plates were imaged with a camera to quantify the colonies.

### Apoptosis detection

Cell apoptosis was analyzed using the FITC Annexin V Apoptosis Detection Kit (BD Biosciences, USA). Collected cells were washed with cold PBS and resuspended in 100 μL of binding buffer, followed by incubation with 5 μL FITC Annexin V and 5 μL PI in the dark for 15 min. Subsequently, 400 μL of binding buffer was added, and samples were analyzed on a BD Influx Cell Sorter (BD Biosciences, USA), with data processed using FACS software.

### Co-immunoprecipitation (Co-IP)

Cells were lysed in RIPA buffer supplemented with a protease and phosphatase inhibitor cocktail (Solarbio). The cell lysates were incubated overnight with primary antibodies against PCMT1 (PA5-110082, ThermoFisher) and LITAF (PA5-22004, ThermoFisher). Protein G agarose beads (Beyotime, China) were then added, and the incubation was continued for 2 h at 4 °C. The beads were collected and washed five times with lysis buffer, and the IP products were subsequently eluted from the beads. The interaction between PCMT1 and LITAF, as well as the ubiquitination level of PCMT1, were evaluated by immunoblotting of the IP products.

### Protein stability assay

To evaluate protein stability, cells exposed to cycloheximide (CHX, 50 μg/mL, Selleck) were harvested at the indicated time points (0, 2, 4, and 8 h), followed by cell lysis and immunoblotting. Protein band intensities were quantified using ImageJ.

### Analysis of PCMT1 ubiquitination site

To identify the specific PCMT1 ubiquitination site mediated by LITAF, a series of point mutations was introduced into a Flag-tagged PCMT1 expression plasmid. The lysine (K) residues at the predicted sites K105, K113, K197, and K206 were mutated to arginine (R) to generate the K105R, K113R, K197R, and K206R mutants. MDA-MB-231/PTX cells were co-transfected with PCMT1-WT or the respective mutants together with HA-Ub and Myc-LITAF. To evaluate PCMT1 ubiquitination, total cell lysates were immunoprecipitated with an anti-Flag antibody (ab205606, Abcam), followed by immunoblotting to detect HA (ab236632, Abcam) and Flag.

### Statistical analysis

All data are presented as mean ± SD (n ≥ 3) and were analyzed using GraphPad Prism 9.0. An unpaired Student’s t-test was applied for comparisons between two groups, whereas one-way ANOVA with Tukey’s multiple comparisons test was used to determine statistical significance among multiple groups. Correlations between PCMT1 and COX-2 expression, as well as between LITAF and PCMT1 expression, were evaluated using the Pearson correlation coefficient. Statistical significance was defined as a P value <0.05.

## Results

### PCMT1 knockdown suppressed COX-2-mediated AA metabolism in BC cells

**FIGURE 1:**
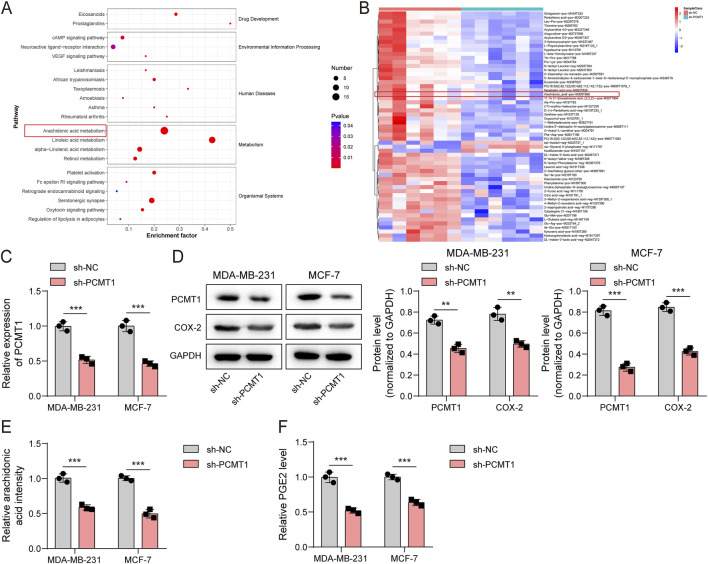
PCMT1 knockdown suppressed the COX-2-mediated AA metabolism in BC cells. **(A,B)** KEGG pathway analysis and differential metabolite analysis of untargeted metabolomics in BC cells (MDA-MB-231) with PCMT1 knockdown (n = 6). **(C)** Knockdown efficiency of PCMT1 in BC cells (MDA-MB-231 and MCF-7) (n = 3). **(D)** Immunoblotting analysis of PCMT1 and COX-2 protein levels (n = 3). **(E,F)** AA and PGE2 levels measured using ELISA kits (n = 3). Values are represented as mean ± SD. *p < 0.05, **p < 0.01, ***p < 0.001.

Knockdown of PCMT1 in MDA-MB-231 cells and subsequent untargeted metabolomic analysis revealed its impact on cellular metabolism. KEGG pathway analysis specifically implicated the AA metabolic pathway in PCMT1-mediated regulation of BC progression (Figure 1A). Consistent with this, differential metabolite analysis showed that PCMT1 inhibition markedly reduced AA levels (Figure 1B). Given this link, we assessed the effect of PCMT1 on AA metabolism in BC cell lines (MDA-MB-231 and MCF-7). Inhibition of PCMT1 noticeably decreased the expression of COX-2 —a key enzyme in AA metabolism—as well as the levels of AA and its major metabolite PGE2 (Figures 1C–F). These results indicate that PCMT1 plays a crucial role in COX-2–regulated AA metabolism in BC cells.

### PCMT1 confers PTX resistance in BC cells, potentially through the COX-2-mediated AA metabolism

**FIGURE 2:**
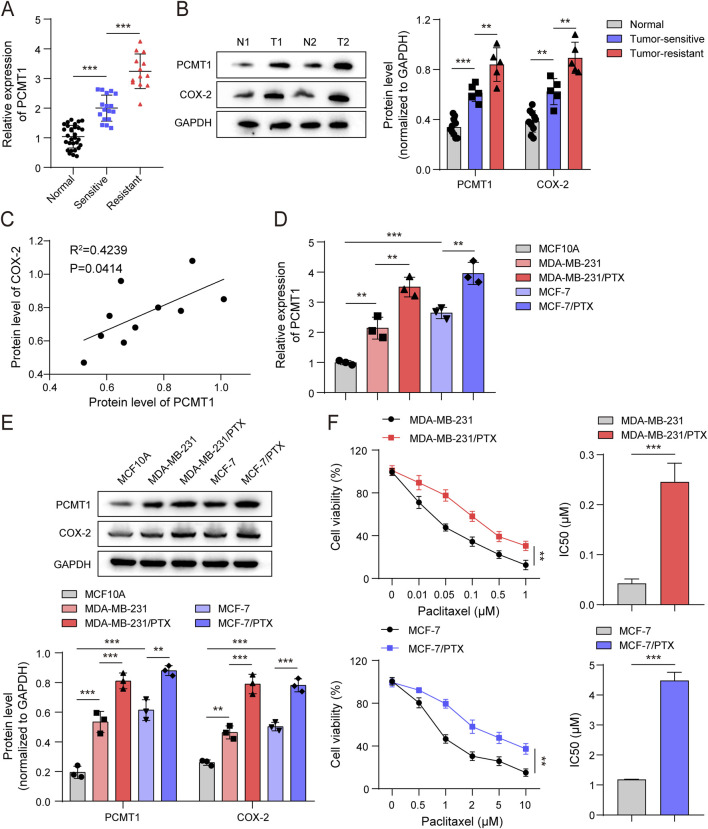
PCMT1 is involved in PTX resistance of BC cells, a process potentially involving COX-2-mediated AA metabolism. **(A)** RT-qPCR analysis of PCMT1 mRNA levels in tumor tissues and paired adjacent normal tissues from 30 BC patients treated with PTX. **(B)** Immunoblotting analysis of PCMT1 and COX-2 protein levels in tumor tissues and paired adjacent normal tissues from PTX-treated BC patients (n = 10; 5 PTX-sensitive and 5 PTX-resistant). **(C)** Correlation between PCMT1 and COX-2 expression was assessed using the Pearson correlation coefficient. **(D)** RT-qPCR analysis of PCMT1 mRNA levels in MCF10A, BC cells (MDA-MB-231, MCF-7), and PTX-resistant cells (MDA-MB-231/PTX, MCF-7/PTX) (n = 3). **(E)** Immunoblotting analysis of PCMT1 and COX-2 protein levels in BC cells and PTX-resistant cells (n = 3). **(F)** IC50 values of BC cells and PTX-resistant cells assessed using the CCK-8 assay (n = 3). Values are represented as mean ± SD. *p < 0.05, **p < 0.01, ***p < 0.001.

Since PCMT1 has been implicated in PTX resistance, we next asked whether PCMT1-regulated AA metabolism contributes to PTX resistance in BC. RT-qRCR and Immunoblotting analysis of clinical specimens revealed that PCMT1 was upregulated in tumor tissues, with visibly higher expression in PTX-resistant cases (Figures 2A,B). Notably, the protein levels of PCMT1 and COX-2 were positively correlated in tumor tissues (Figure 2C), linking PCMT1 to the COX-2–mediated AA metabolic pathway in the context of resistance. To functionally test this link, we established PTX-resistant cell lines (MDA-MB-231/PTX and MCF-7/PTX). Consistent with the clinical data, both PCMT1 and COX-2 were markedly upregulated in these resistant cells (MDA-MB-231/PTX and MCF-7/PTX) compared to their parental counterparts (MDA-MB-231 and MCF-7) and to normal breast epithelial MCF10A (Figures 2D,E). As expected, the resistant cells exhibited significantly higher IC50 values for PTX (Figure 2F), confirming their resistant phenotype. Based on these findings, subsequent experiments utilized PTX at 0.25 μM and 4.50 μM for MDA-MB-231/PTX and MCF-7/PTX cells, respectively. Collectively, these data suggest that PCMT1-regulated AA metabolism, potentially through COX-2, is associated with PTX resistance in BC cells.

### PCMT1 inhibition restrained BC proliferation and enhanced the sensitivity of BC cells to PTX via modulating AA pathway

**FIGURE 3:**
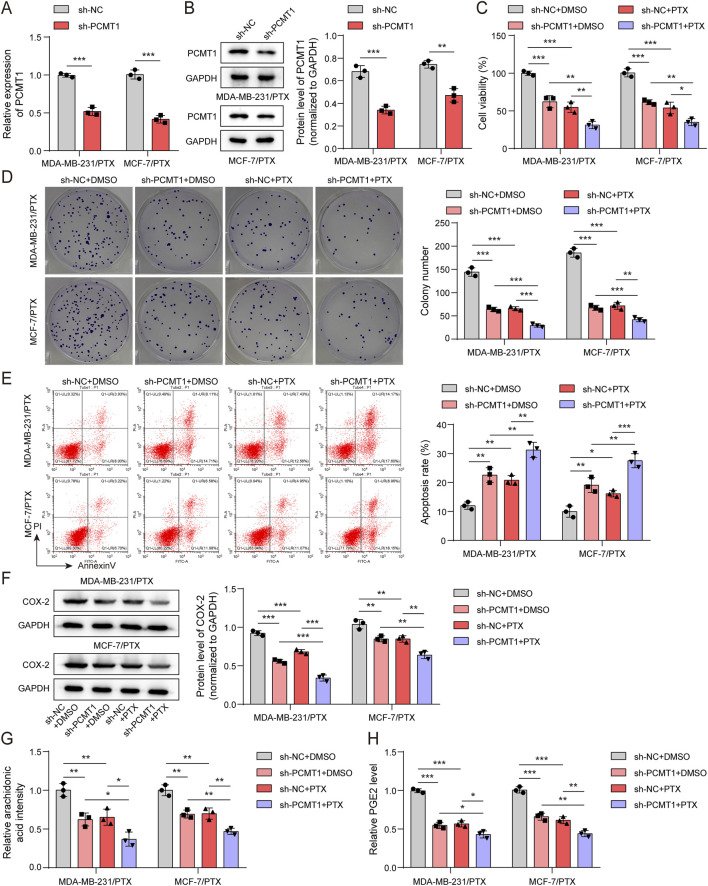
Inhibition of PCMT1 restrained BC proliferation and enhanced BC cells' sensitivity to PTX via modulating the AA pathway. Inhibition of PCMT1 combined with PTX treatment in MDA-MB-231/PTX and MCF-7/PTX cells (n = 3). **(A,B)** PCMT1 mRNA and protein levels were assessed using RT-qPCR and immunoblotting, respectively. **(C,D)** Cell viability and proliferative capacity were evaluated using the CCK-8 assay and colony formation assay, respectively. **(E)** Cell apoptosis was analyzed using the FITC Annexin V Apoptosis Detection Kit. **(F)** COX-2 protein levels were examined by immunoblotting. **(G,H)** AA and PGE2 levels were measured using ELISA kits. Values are represented as mean ± SD. *p < 0.05, **p < 0.01, ***p < 0.001.

To validate whether PCMT1 is required for maintaining PTX resistance, we then inhibited PCMT1 in the PTX-resistant cell lines MDA-MB-231/PTX and MCF-7/PTX, both with and without PTX treatment. RT-qPCR and immunoblotting data displayed that sh-PCMT1 treatment caused an observable decrease in PCMT1 mRNA and protein levels, indicating successful knockdown of PCMT1 in PTX-resistant cells (Figures 3A,B). Strikingly, the CCK-8 and clonogenic assays showed that PCMT1 knockdown was sufficient to impair cell viability and colony formation, and it synergistically enhanced the cytotoxic effects of PTX in these resistant cells (Figures 3C,D). Additionally, flow cytometry revealed that PCMT1 depletion promoted the apoptosis of PTX-resistant cells and augmented the pro-apoptotic effect of PTX (Figure 3E). Immunoblotting and ELISA results demonstrated that PCMT1 inhibition diminished the levels of COX-2, AA, and PGE2 in PTX-resistant cells and potentiated the suppressive effect of PTX on this metabolic pathway (Figures 3F–H). These results indicate that knockdown of PCMT1 suppresses the AA pathway, which correlates with reduced cell proliferation and restored sensitivity to PTX.

### LITAF interacted with PCMT1 and mediated the ubiquitination-dependent degradation of PCMT1

**FIGURE 4:**
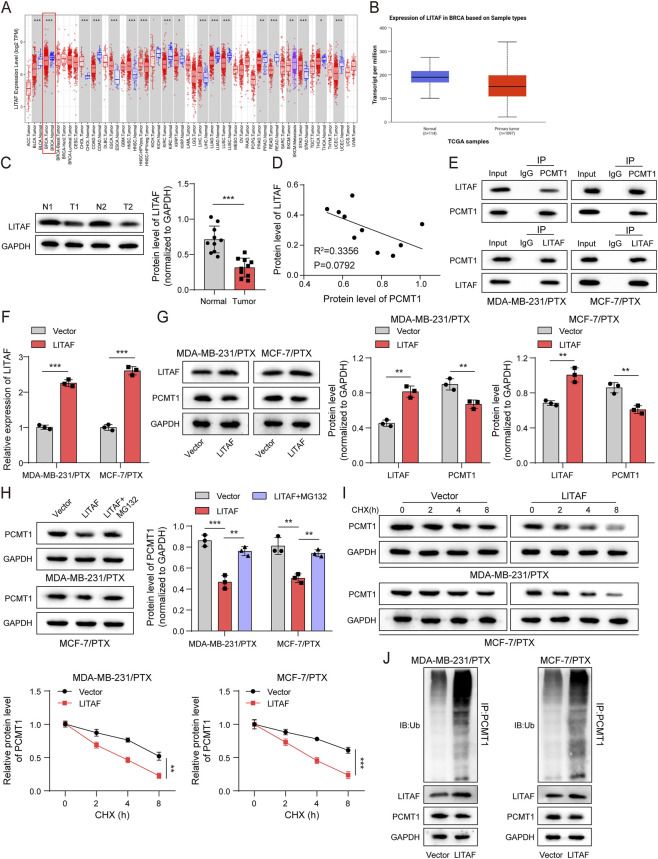
LITAF interacted with PCMT1 and mediated PCMT1’s ubiquitination-dependent degradation. **(A,B)** LITAF expression in BC was analyzed using TIMER and TCGA databases. **(C)** Immunoblotting was performed to detect LITAF protein expression in tumor tissues (n = 10). **(D)** Correlations between LITAF and PCMT1 expression were assessed using the Pearson correlation coefficient. **(E)** The interaction between LITAF and PCMT1 was examined by Co-IP in PTX-resistant cells (n = 3). Overexpression of LITAF in PTX-resistant cells (n = 3). **(F)** RT-qPCR confirmed the mRNA level of LITAF. **(G)** Immunoblotting assessed the protein levels of LITAF and PCMT1. **(H)** Immunoblotting evaluated PCMT1 protein levels after MG132 treatment. **(I)** Immunoblotting evaluated PCMT1 protein levels after CHX treatment. **(J)** IP followed by immunoblotting was performed to assess PCMT1 ubiquitination. Values are represented as mean ± SD. *p < 0.05, **p < 0.01, ***p < 0.001.

To elucidate the upstream regulation of PCMT1, we employed the Ubibrower2.0 database and identified the E3 ligase LITAF as a potential regulator. Analyses of public datasets (TIMER, TCGA) and our own cohort consistently showed that LITAF expression was significantly downregulated in BC tissues (Figures 4A–C). Furthermore, LITAF levels were inversely correlated with PCMT1 expression (Figure 4D). Then, Co-IP assays were performed using lysates from PTX-resistant cells to explore PCMT1 and LITAF’s binding. PCMT1 was found to co-precipitate with LITAF when using an anti-LITAF antibody, and conversely, LITAF co-precipitated with PCMT1 when using an anti-PCMT1 antibody, confirming a physical interaction between the two proteins (Figure 4E). Functionally, overexpression of LITAF in PTX-resistant cells led to a marked reduction in PCMT1 protein levels (Figures 4F,G), which was rescued by the proteasome inhibitor MG132 (Figure 4H). Cycloheximide chase assays further demonstrated that LITAF accelerated the degradation of PCMT1 protein (Figure 4I). Consistently, LITAF overexpression enhanced the ubiquitination of PCMT1 (Figure 4J).

We next sought to identify the ubiquitination sites on PCMT1 that are modified by LITAF. Based on these predicted sites, we generated a series of lysine-to-arginine point mutants (K105R, K113R, K197R, K206R) in PCMT1. Ubiquitination assay revealed that mutations at sites 197 and 206, but not 105 and 113 abolished LITAF-mediated ubiquitination of PCMT1 (Supplementary Figure S1).

Taken together, these data demonstrate that LITAF facilitates the ubiquitin-mediated proteasomal degradation of PCMT1, effectively reducing its expression.

### LITAF modulated PCMT1-mediated AA metabolism to suppress the proliferation of PTX-resistant BC cells

**FIGURE 5:**
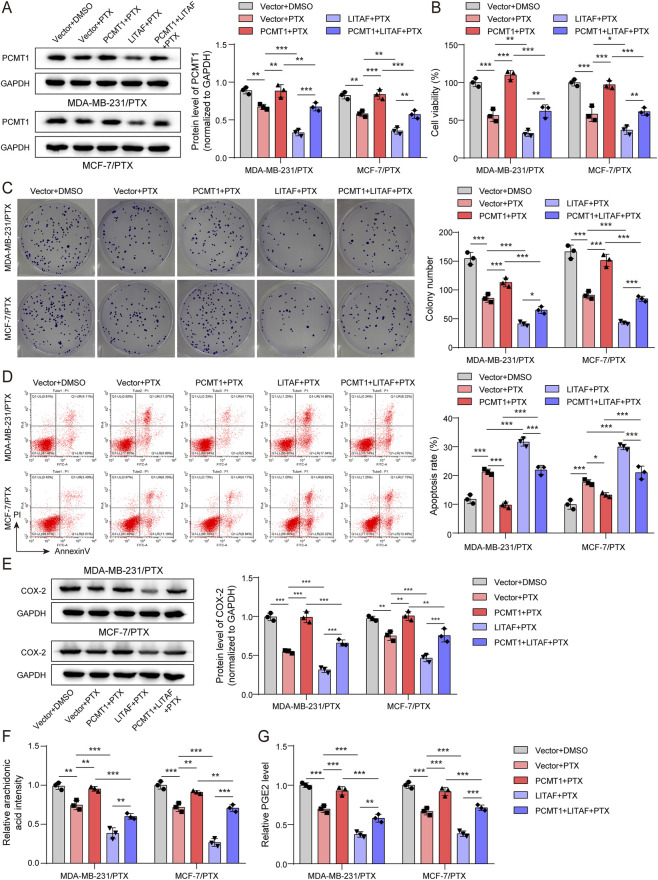
LITAF modulated PCMT1-mediated AA metabolism to suppress the proliferation of PTX-resistant cells. Overexpression of LITAF/PCMT1 and PTX treatment in PTX-resistant cells (n = 3). **(A)** PCMT1 protein levels were examined using immunoblotting. **(B,C)** Cellular proliferation was evaluated using the CCK-8 assay and colony formation assay, respectively. **(D)** Cell apoptosis was monitored using the FITC Annexin V Apoptosis Detection Kit. **(E)** COX-2 protein levels were assessed by immunoblotting. **(F,G)** AA and PGE2 levels were measured using ELISA kits. Values are represented as mean ± SD. *p < 0.05, **p < 0.01, ***p < 0.001.

Having established that LITAF promotes PCMT1 degradation, we next asked whether they function antagonistically to regulate PTX resistance. Immunoblotting analysis revealed that LITAF overexpression reduced PCMT1 levels, while PCMT1 overexpression increased them and counteracted the effect of LITAF (Figure 5A). In functional assays, PCMT1 overexpression significantly attenuated the anti-proliferative and pro-apoptotic effects of PTX, as evidenced by increased cell viability, colony formation, and reduced apoptosis (Figures 5B–D). Conversely, LITAF overexpression synergized with PTX, further suppressing proliferation and promoting apoptosis. Critically, the co-overexpression of PCMT1 rescued the cells from the sensitizing effects of LITAF, restoring PTX resistance (Figures 5B–D). Additionally, PCMT1 overexpression counteracted PTX-induced downregulation of COX-2, AA, and PGE2 in PTX-resistant cells, whereas LITAF overexpression synergistically enhanced this suppression (Figures 5E–G). Co-expression of PCMT1 reversed the inhibitory effect of LITAF on this pathway (Figures 5E–G). These rescue experiments demonstrate that LITAF sensitizes BC cells to PTX by degrading PCMT1, thereby inhibiting the AA metabolic pathway.

### LITAF suppressed the growth of BC and enhanced their sensitivity to PTX *in vivo*

**FIGURE 6:**
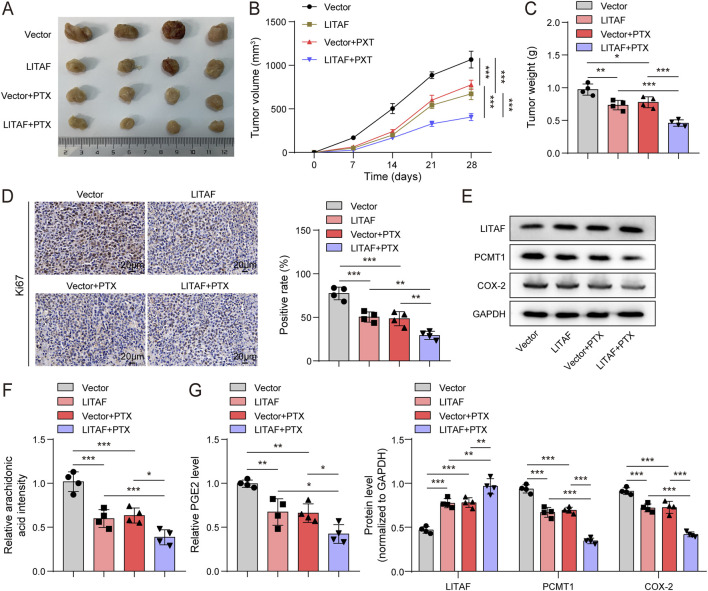
LITAF suppressed the growth of BC and enhanced their sensitivity to PTX *in vivo*. MDA-MB-231/PTX cells stably expressing LITAF were inoculated subcutaneously into nude mice, and the mice were then treated with PTX starting from day 7 (n = 4). **(A)** Representative photograph of the excised tumors. **(B)** Tumor volume on days 7, 14, 21, and 28. **(C)** Tumor weight at the end of the experiment. **(D)** IHC tested the expression of Ki67 in tumor tissues; Images were acquired at ×400 magnification (scale bars, 20 µm). **(E)** Immunoblotting tested the protein levels of LITAF, PCMT1, and COX-2 in tumor tissues. **(F,G)** AA and PGE2 levels were monitored using ELISA kits. Values are represented as mean ± SD. *p < 0.05, **p < 0.01, ***p < 0.001.

To further validate the *in vivo* role of the LITAF/PCMT1/COX-2 axis, we generated subcutaneous xenografts from MDA-MB-231/PTX cells stably overexpressing LITAF and treated the tumor-bearing mice with PTX. PTX treatment significantly inhibited tumor growth, and this inhibition was markedly potentiated by LITAF overexpression (Figures 6A–C), indicating enhanced tumor sensitivity to PTX. Aligned with this, IHC results showed that treatment with PTX observably reduced Ki67 expression, with tumors from LITAF-overexpressing mice showing even lower Ki67-positive rates upon PTX administration (Figure 6D). Furthermore, immunoblotting and ELISA results showed that PTX downregulated the levels of PCMT1, COX-2, AA, and PGE2 in tumors and increased LITAF levels, an effect that was more pronounced in LITAF-overexpressing groups (Figures 6E–G). These results proved that LITAF overexpression potentiates PTX’s ability to suppress tumor growth and inhibit the PCMT1/COX-2 pathway.

## Discussion

PTX is one of the main chemotherapeutic agents used in the treatment of BC, but many patients develop drug resistance during the course of therapy, which ultimately results in chemotherapy failure and poor prognosis (Wu et al., 2024; Fu et al., 2024). In this study, we found that inhibition of PCMT1 disrupts COX-2-mediated AA metabolism and increases the sensitivity of PTX-resistant BC cells to PTX. Furthermore, LITAF overexpression suppresses PCMT1, thereby further inhibiting COX-2-mediated AA metabolism and enhancing the sensitivity of PTX-resistant BC cells to PTX, as well as increasing the responsiveness of tumors to PTX *in vivo*.

PCMT1 acts as an oncogenic driver in the progression of various cancers, including BC (Rogaczewski et al., 2025; Zhang et al., 2022; Peng et al., 2025). In the present study, untargeted metabolomics revealed that PCMT1 expression was associated with alterations in AA metabolism. COX-2-mediated AA metabolism plays a critical role in cancer progression and in the development of tumor drug resistance (Saito et al., 2021), and one study reported that COX-2 is upregulated in chemotherapy-treated cells and is linked to enhanced tumor invasion and reduced apoptosis (Khan et al., 2011). Consistent with this, our data showed that inhibition of PCMT1 attenuated COX-2-mediated AA metabolism in BC cells, as reflected by decreased levels of COX-2, AA, and PGE2. In tumor samples from BC patients, PCMT1 expression was positively correlated with COX-2 expression. Previous research has reported that silencing PCMT1 enhances BC cells' sensitivity to PTX via the Akt/STMN1 pathway (Zhang et al., 2024), and our findings are in line with this observation, as PCMT1 expression levels were higher in tumor tissues from PTX-resistant BC patients than in those from PTX-sensitive patients. Similarly, PTX-resistant BC cells exhibited higher levels of PCMT1 and COX-2 compared with their parental counterparts. Knockdown of PCMT1 in PTX-resistant cells increased their sensitivity to PTX treatment, as evidenced by reduced cell growth and enhanced apoptosis, and was accompanied by a reduction in COX-2-mediated AA metabolism.

LITAF is an E3 ubiquitin ligase that mediates the ubiquitination and degradation of downstream substrates (Min et al., 2025), and in tumor tissues from BC patients, we observed an inverse correlation between the expression of LITAF and PCMT1. Functionally, consistent with its role as an E3 ubiquitin ligase, LITAF bound to PCMT1 and mediated its ubiquitin-dependent degradation through lysine residues 197 and 206. LITAF has been implicated in the regulation of cancer cell proliferation, apoptosis, migration, and stemness (Guan et al., 2023). Huang C et al. demonstrated that LITAF enhances the radiosensitivity of human glioma cells via the FOXO1 pathway (Huang et al., 2019). Hoey C et al. found that low LITAF expression is associated with radiotherapy resistance in prostate cancer (Hoey et al., 2018). In our cohort, LITAF was downregulated in tumor tissues from BC patients, and LITAF overexpression reduced PCMT1 expression, thereby inhibiting COX-2-mediated AA metabolism, increasing the sensitivity of PTX-resistant BC cells to PTX, and strengthening the inhibitory effect of PTX on tumor growth *in vivo*.

In conclusion, our data demonstrate that LITAF inhibits COX-2-mediated AA metabolism by promoting the ubiquitination-mediated degradation of PCMT1, thereby enhancing BC cells' sensitivity to PTX and potentiating the ability of PTX to suppress tumor growth *in vivo*. These findings not only provide new diagnostic and therapeutic targets for BC, particularly PTX-resistant disease, but also support a novel treatment strategy in which PTX is combined with interventions targeting the LITAF/PCMT1/COX-2 pathway to overcome PTX resistance and enhance chemotherapeutic efficacy in BC.

## Data Availability

The raw data supporting the conclusions of this article will be made available by the authors, without undue reservation.
