# Supplementary figures and images for: LITAF suppresses breast cancer and paclitaxel resistance by ubiquitinating and degrading PCMT1 to inhibit COX-2-dependent arachidonic acid metabolism
Source: Front Pharmacol. 2026 Apr 13;17:1706420. doi: 10.3389/fphar.2026.1706420 (PMC13111324; doi:10.3389/fphar.2026.1706420)

Supplementary material-uncropped original western blots


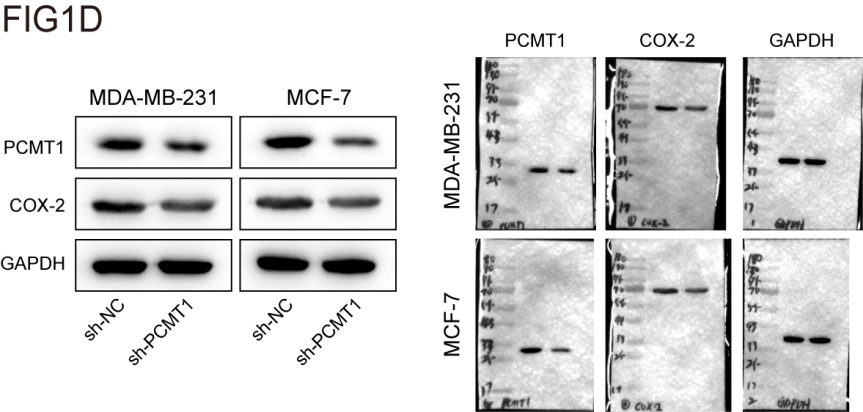


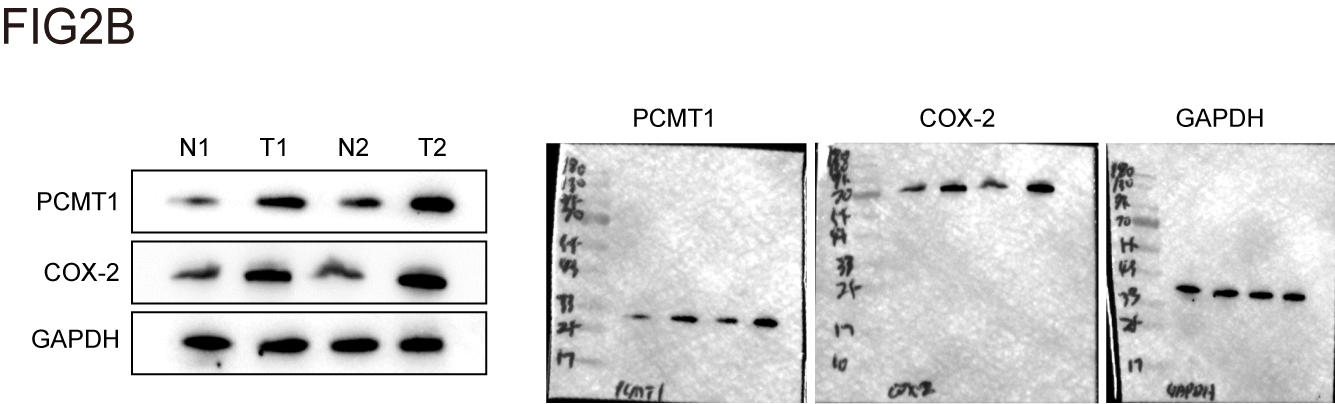


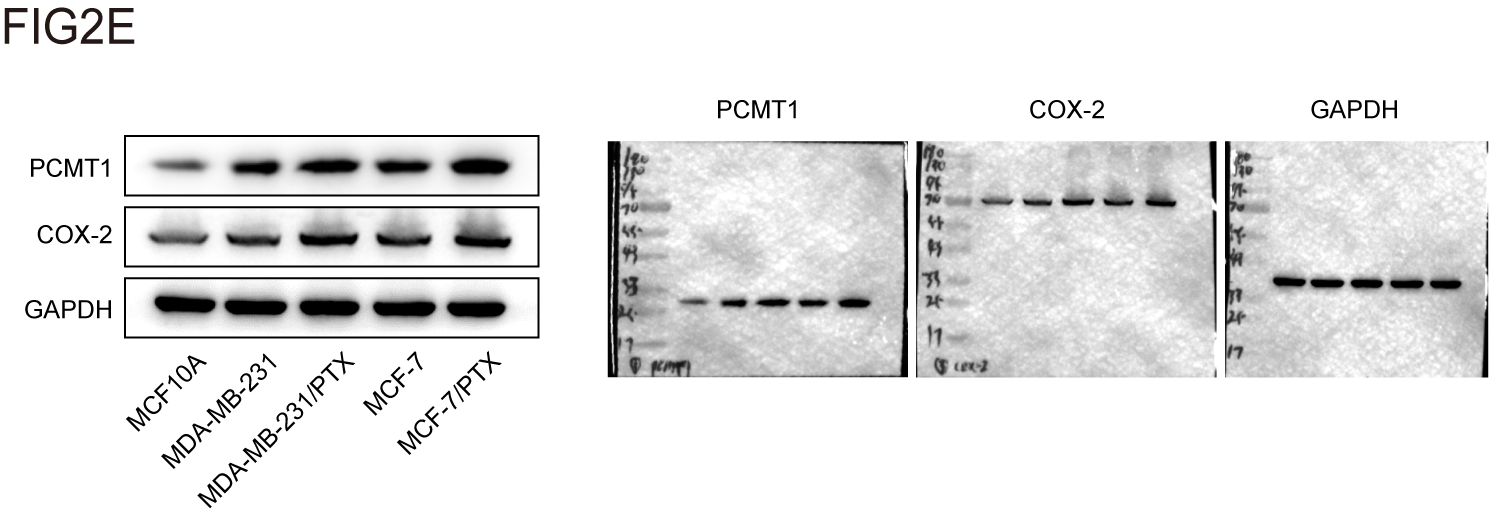


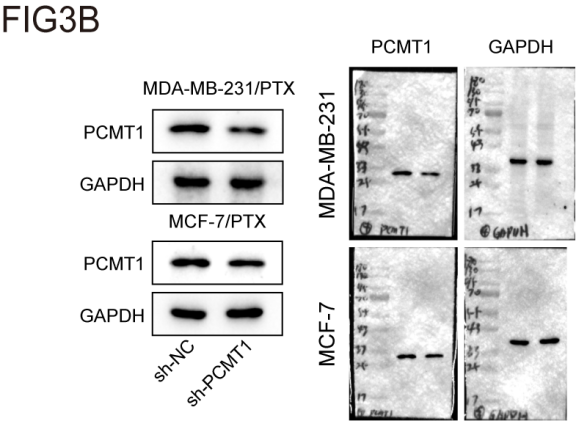


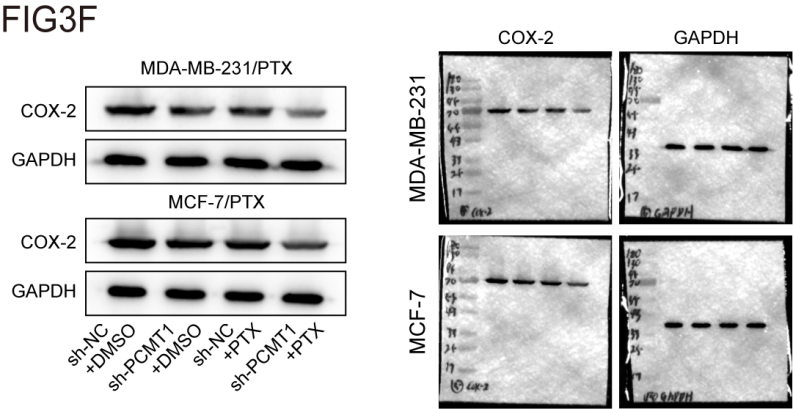


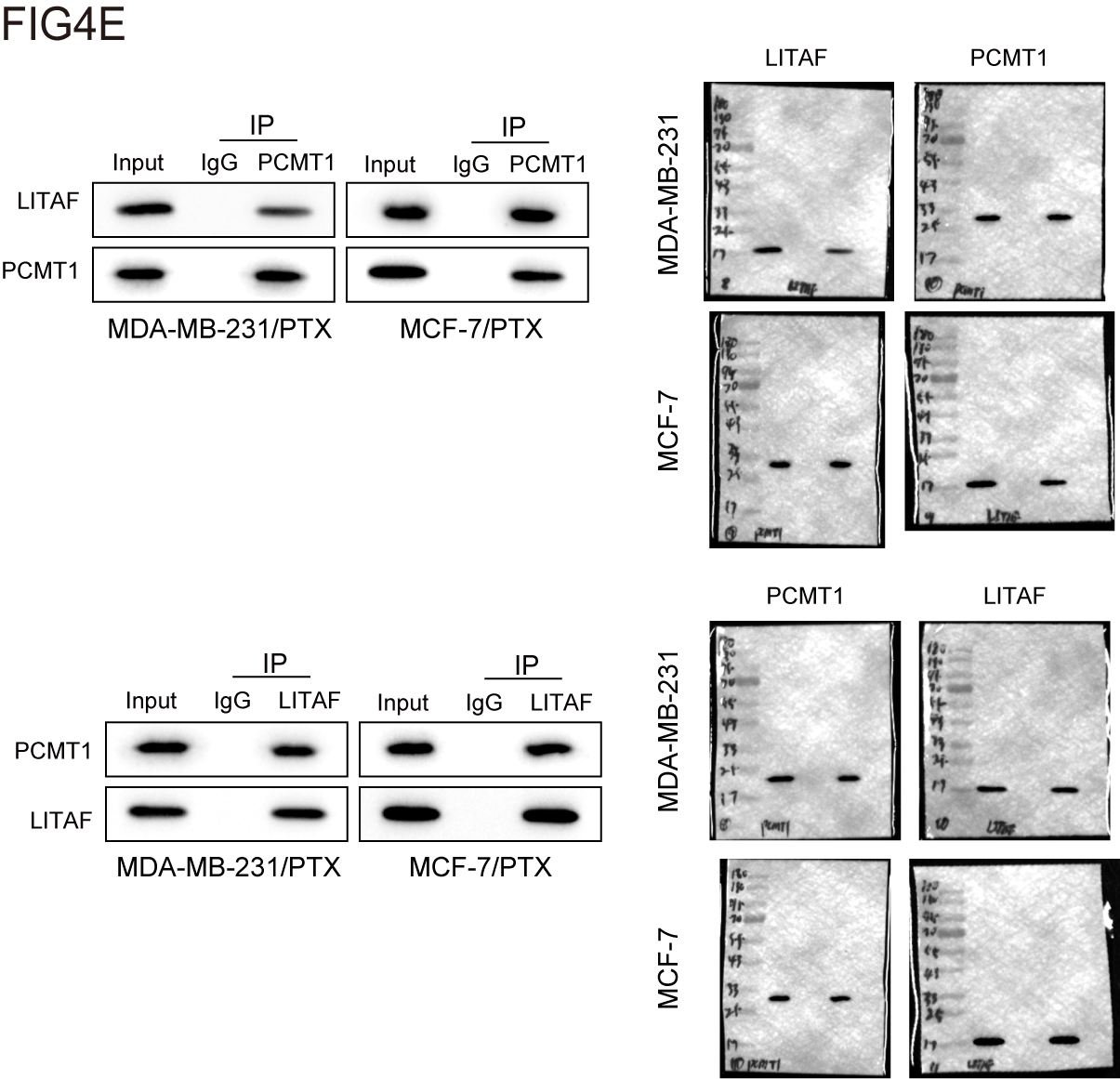


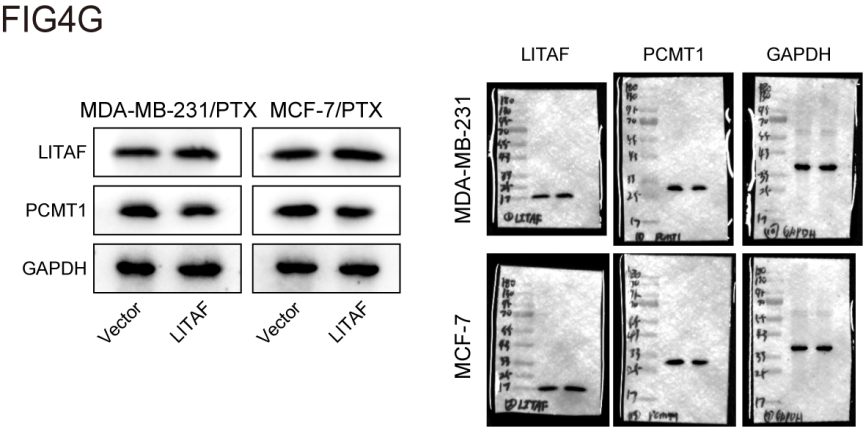


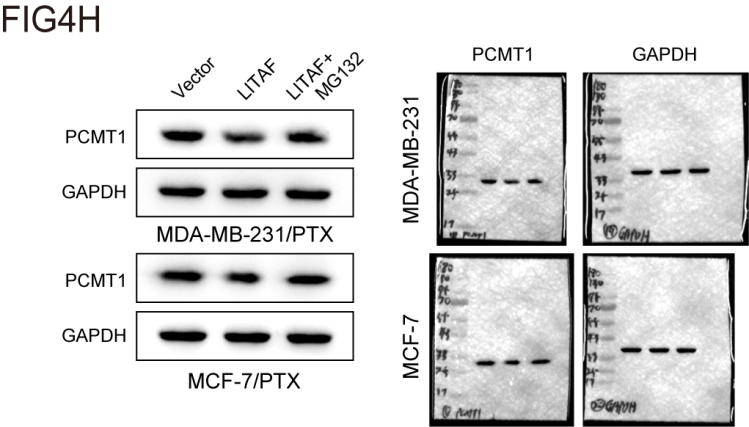


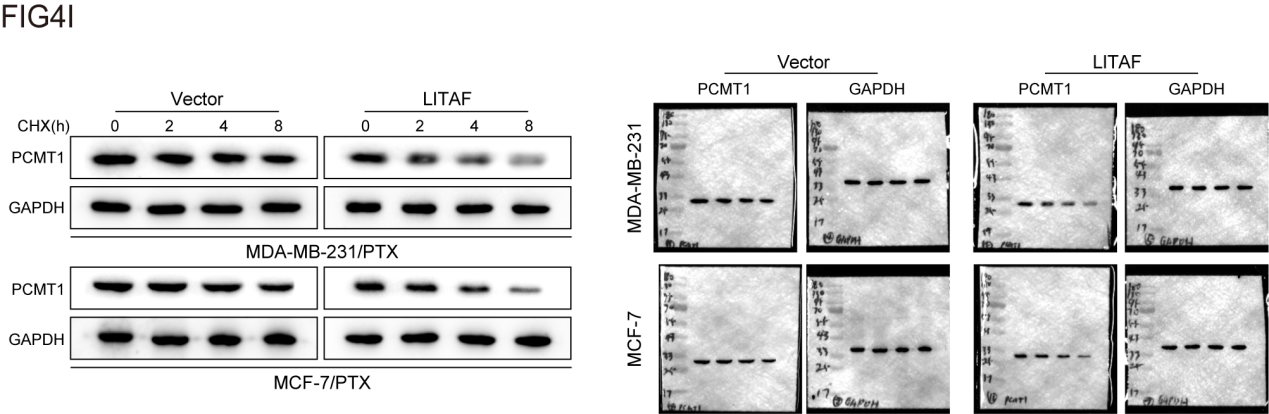


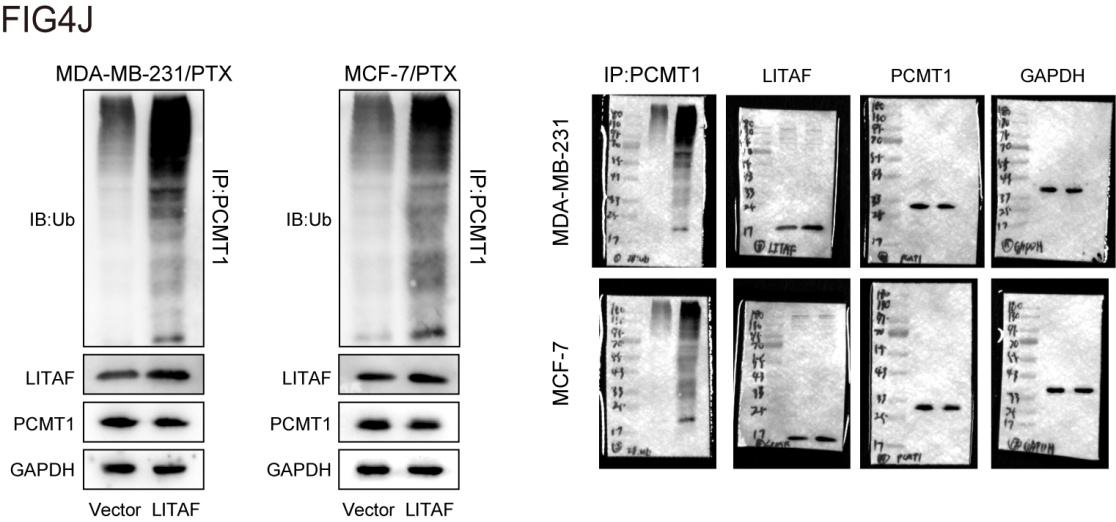


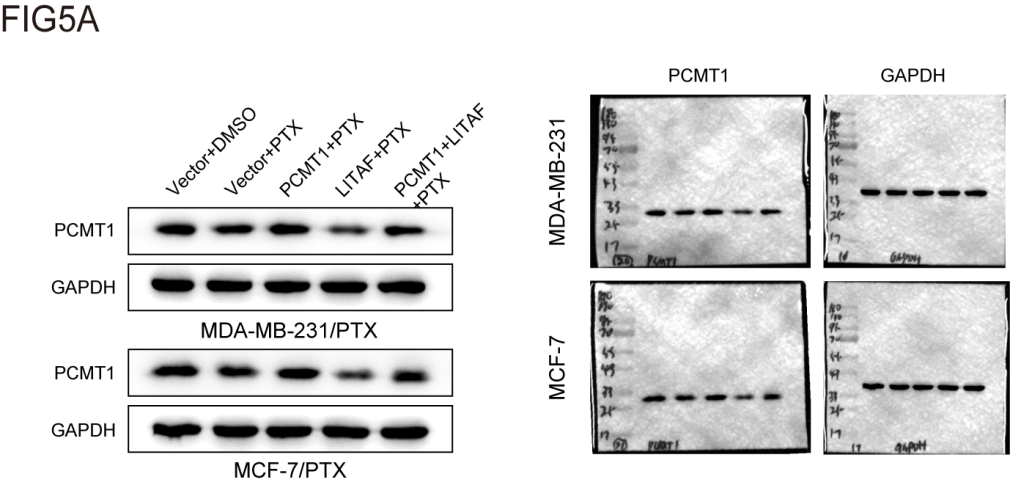


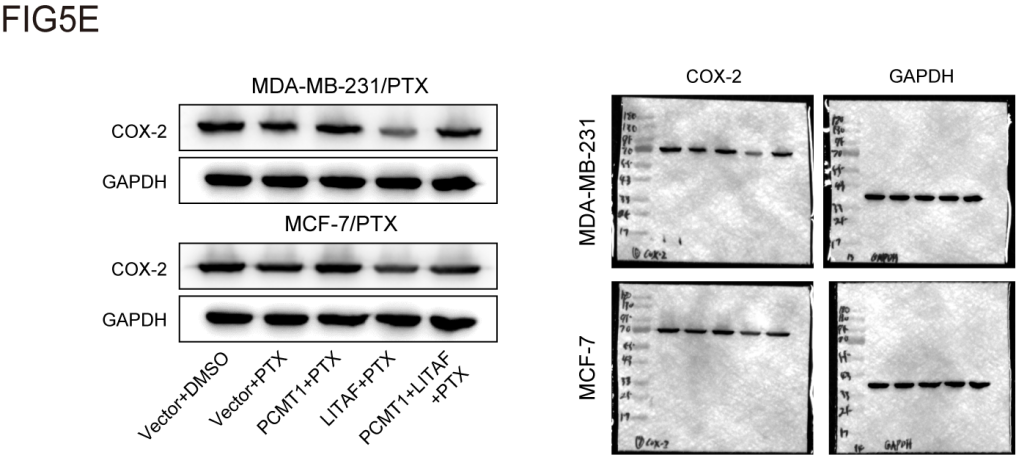


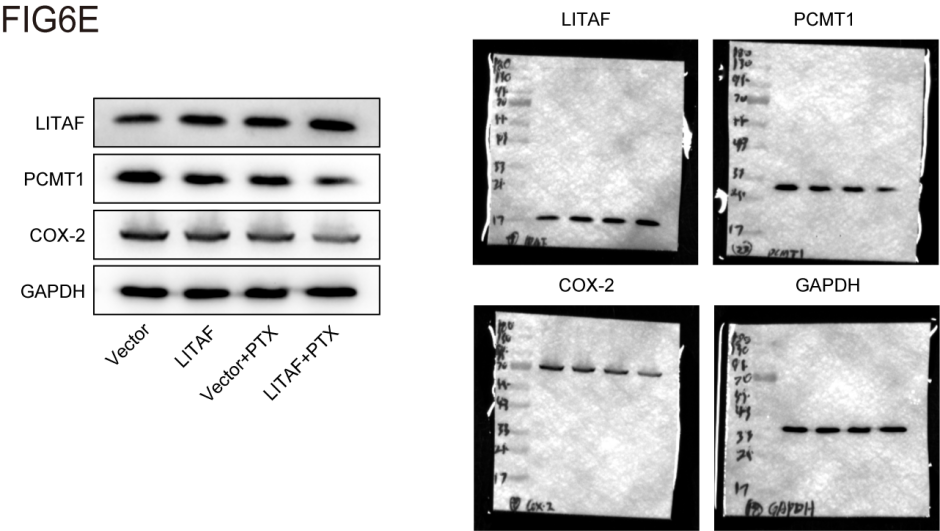

Supplement: Supplementary file 1 [file DataSheet1.docx]
